# Orbital Apex Syndrome in Herpes Zoster Ophthalmicus

**DOI:** 10.1155/2012/854503

**Published:** 2012-07-09

**Authors:** Hatice Arda, Ertugrul Mirza, Koray Gumus, Ayse Oner, Sarper Karakucuk, Ender Sırakaya

**Affiliations:** Department of Ophthalmology, Medical Faculty Erciyes University, 38039 Kayseri, Turkey

## Abstract

Orbital apex syndrome is a rare manifestation of Herpes Zoster Ophthalmicus. Herein we report on a case of orbital apex syndrome secondary to Herpes Zoster Ophthalmicus. A 75 year-old male complained of vision loss, conjunctival hyperemia and proptosis on the left eye, was referred to our clinic. Visual acuity was 5/10 Snellen lines and he had conjunctival hyperemia, chemosis, minimal nuclear cataract and proptosis on the left eye. A diagnosis of orbital pseudotumor was demonstrated firstly. The patient received oral and topical corticosteroids, antiinflammatory and antibiotic agents. On day 2, vesiculopustular lesions were observed, Herpes Zoster Ophthalmicus was diagnosed and corticosteroid treatment stopped, oral acyclovir treatment initiated. Two days later, total ophthalmoplegia, ptosis and significant visual loss were observed on the left. The diagnosis of orbital apex syndrome was considered and the patient commenced on an intravenous acyclovir treatment. After the improvement of acute symptoms, a tapering dose of oral cortisone treatment initiated to accelarate the recovery of ophthalmoplegia. At 5-month follow-up, ptosis and ocular motility showed improvement. VA did not significantly improve because of cataract and choroidal detachment on the left. We conclude that ophthalmoplegia secondary to Herpes Zoster Ophthalmicus responds favourably to intravenous acyclovir and steroids.

## 1. Introduction

Herpes zoster is a localized disease characterised by unilateral radicular pain and a vesicular eruption caused by varicella zoster virus which is a human neurotropic DNA virus [1, 2]. Orbital involvement with HZO includes keratoconjunctivitis, anterior uveitis, acute retinal necrosis, acute phthisis bulbi, central retinal artery occlusion, optic neuritis, orbital pseudotumor, and partial or complete paralysis of ocular motility [3–6]. The extraocular muscle palsies occur in 3.5–10.1% of patients with ophthalmic zoster; these are transient, self-limited, and usually seen in the elderly [4, 5, 7, 8]. The orbital apex syndrome (OAS) is defined by the association of visual loss, ophthalmoplegia, blepharoptosis, proptosis, and anesthesia of the upper eyelid and forehead. It is a rare manifestation of HZO [9]. In this study we report on a case of orbital apex syndrome secondary to HZO.

## 2. Case Report

A 75-year-old male referred to the eye clinic of Erciyes University Medical Faculty with the complaint of vision loss and conjunctival hyperemia of the left eye. He had a history of trauma to his left eye four days ago. Magnetic resonance imaging (MRI) of the orbits showed bilateral proptosis which was significant on the left as well as edema and inflammation at the anteroinferior part of the left bulbus oculi. He was referred to our clinic to investigate the etiology of the proptosis. His past medical history included hypertension which was regulated with an oral antihypertensive agent. Visual acuity was counting fingers on the right eye and 5/10 Snellen lines on the left. He had nuclear cataract, and minimal proptosis on the right eye, conjunctival hyperemia, chemosis, nuclear cataract and moderate proptosis on the left eye. The intraocular pressure and fundus examinations were within normal limits for both eyes. Hertel exophthalmometer measurements were 21 mm on the right and 24 mm on the left. Ocular ultrasonographic (USG) examination of both eyes were normal. The patient was hospitalized for the differential diagnosis of orbital pseudotumor, orbital cellulitis, retrobulbar tumor, intraorbital foreign body, thyroid ophthalmopathy, and carotid-cavernous fistula. The patient received oral flurbiprofen (100 mg, t.i.d) and ciprofloxacin (750 mg, b.i.d), topical moxifloxacine and dexamethasone 6 times daily and cold compresses as an initial treatment. Orbital computerized tomography showed no foreign body, and there was no murmur on auscultation of the glob. The proptosis was not pulsatile. The visual acuity on the left eye decreased to 4/10 Snellen lines one day after the hospitalization although the other ocular signs remained the same. After the consultations with the departments of infectious diseases and endocrinology, the diagnosis of orbital cellulitis and/or thyroid ophthalmopathy was not considered. An oral steroid (fluocortolone 1 mg/kg,) and preventive treatment for the gastric mucosa (ranitidine HCL, b.i.d) were added to the treatment because of the possible diagnosis of orbital pseudotumor.

**Figure 1:**
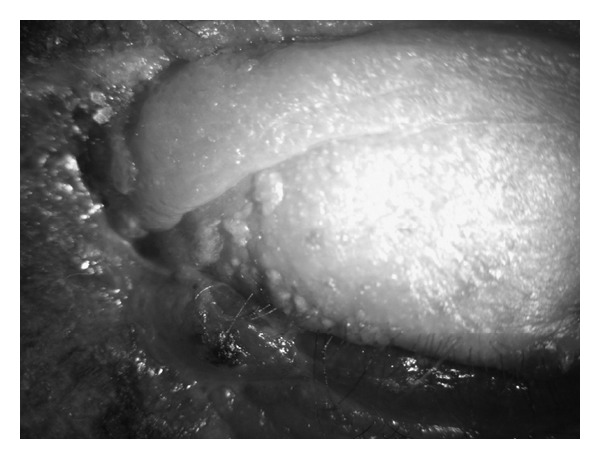
Vesicular lesions on the left upper eyelid.

**Figure 2:**
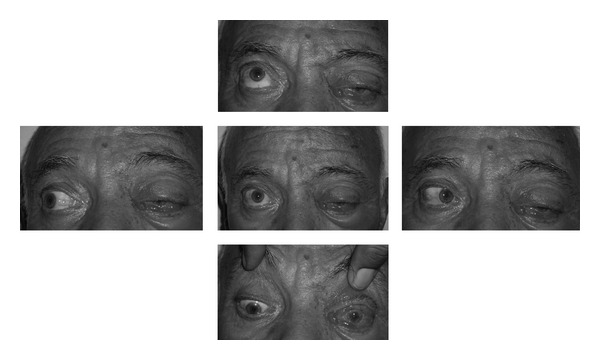
Total ophthalmoplegia and ptosis of the left eye.

**Figure 3:**
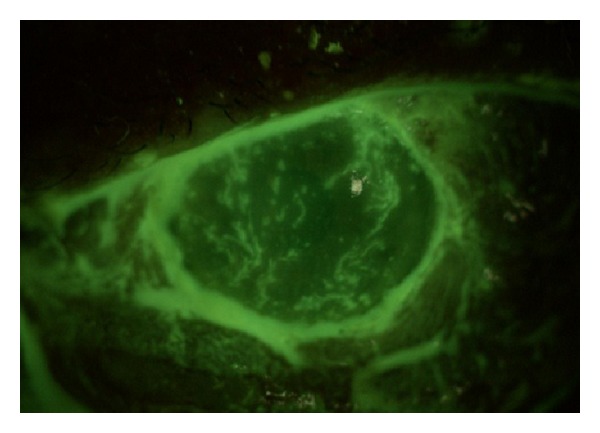
Dendritic lesions on the left cornea.

**Figure 4:**
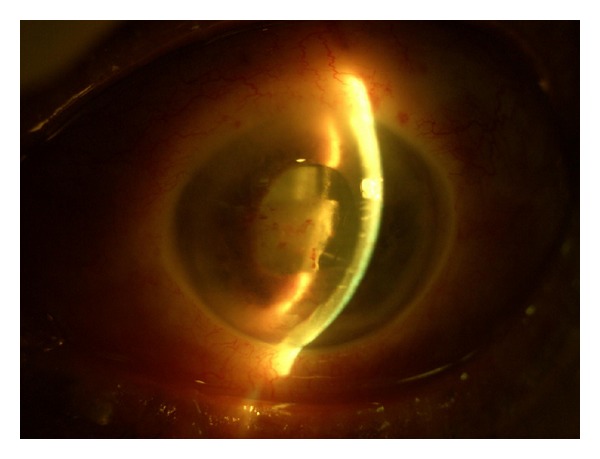
Cyclitic membrane and hemorrhage in the left anterior chamber.

 On the second day of the hospitalization, vesicular and pustular lesions occurred around the left eyelid and forehead, and the diagnosis of HZO was made (Figure 1). The visual acuity of the left eye rapidly decreased to counting fingers from 3 meters, and a punctate keratopathy was detected on slit-lamp examination. After the consultation with the department of dermatology, oral corticosteroid treatment was stopped, and the patient was commenced on a treatment of oral valacyclovir (1 g, t.i.d. for 7 days), vitamin B1 (250 mg, b.i.d), vitamin B6 (250 mg, b.i.d), and dressing with ethacridine lactate for the skin lesions. The visual acuity of the left eye decreased to hand motions one day after the initialization of this treatment. Total ophthalmoplegia and ptosis of the left eye were detected (Figure 2). There were dendritic lesions on the cornea (Figure 3). Since a brief period of disorientation and a syncope was developed, a diagnosis of OAS and a probable cranial involvement was considered and the patient was referred to the department of infectious diseases. The patient was commenced on intravenous ampicillin sulbactam (2 gr, t.i.d.) and acyclovir (750 mg, t.i.d.) for 14 days, topical acyclovir ointment 5 times, moxifloxacin and polyacrylic acid 6 times, and a fixed combination of dorzolamide HCL and timolol maleate 2 times per day. Two days later uveitis with cyclitic membrane and hemorrhage in the anterior chamber were developed (Figure 4). Topical cycloplegic drops and hot eye compresses were added to the treatment 5 times per day. Topical steroids were not considered because of the diffuse corneal epithelial defects. MRI examination showed an enlargement of the extraocular muscles and an increase in the amount of the soft tissues around the preseptal area; both of the cavernous sinuses were normal. Both of the superior orbital veins were dilated, which were more significant on the left. There was a choroidal detachment and an intraocular hemorrhage on the left eye. Diffusion MRI showed a deficiency of the diffusion on the right frontal and frontoparietal regions.

 When the acute clinical signs of HZO were regressed, the patient was commenced on a tapering dose of oral prednisolone (1 mg/kg per day) to accelerate the recovery of the total ophthalmoplegia, proptosis, and ptosis. Topical cortisone treatment (dexamethasone) 8 times was added after the recovery of the corneal epithelial defects.

**Figure 5:**
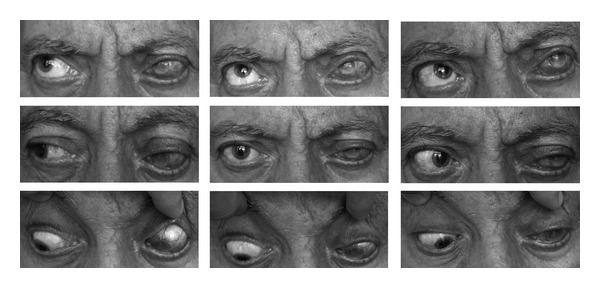
Partial improvement of the ocular motility and ptosis of the left eye after 5-month followup.

 At the end of 5-month follow-up period, the visual acuity of the left eye was counting fingers from 1.5 meters. Anterior segment examination showed an epithelial defect at the inferior half of the cornea, posterior synechiae at the pupillary area, fibrinoid reaction in the anterior chamber and mature cataract. The intraocular pressure of the left eye was 4 mmHg (applanation). The USG examination of the left eye revealed a choroidal detachment. The corneal sensation, ocular motility, and the ptosis showed partial improvement at the end of five months (Figure 5).

## 3. Discussion

HZO is a disease in which the ophthalmic division of the trigeminal nerve is affected by the varicella virus [10]. The common ocular manifestations of HZO include blepharoconjunctivitis, keratitis, and uveitis [11]. OAS is a rare and severe manifestation of HZO which was to the best of our knowledge reported in only five cases previously [9, 12–14]. We reported the sixth case of HZO which progressed to OAS in the literature. OAS is characterized by the involvement of the 2nd, 3rd, 4th, and 6th cranial nerves with the paralysis of ophthalmic branch of the 5th cranial nerve due to inflammatory, infectious, neoplastic, traumatic, vascular, and sometimes iatrogenic reasons along the ophthalmic canal region [15]. In our case there was a history of trauma and a viral infection but not any malignancy. The two of the previously reported cases had immunodeficiency; one had Hodgkin's disease and in the other case, human immunodeficiency virus was positive [12, 14]. All of the cases which had orbital apex syndrome secondary to HZO in the literature were treated with systemic acyclovir and steroids. The treatment with intravenous acyclovir and steroids usually carries a good prognosis with the exception of the presence of immunosuppression. Superior orbital fissure syndrome (SOFS) has the same clinical findings with OAS. The clinical signs of SOFS include proptosis, ptosis, and total ophthalmoplegia similar to orbital apex syndrome. SOFS can be distinguished from the orbital apex syndrome by the absence of optic nerve involvement. The visual acuity of the left eye rapidly decreased in our patient. The appearance of the optic disc was normal during the initial fundus examination. After the development of anterior uveitis and secondary cataract, we were unable to evaluate the optic disc and retina to disclose an optic nerve atrophy.

 The time interval for resolution of ophthalmoplegia secondary to HZO was found between 2 weeks to 1.5 years and a mean of 4.4 months in a previous review report [16]. At the end of the 5-month follow-up period, partial resolution of the ocular movements and ptosis was observed in the presented case. The follow-up period could have been longer to observe the complete resolution; however, our patient went abroad and we were unable to follow him up any longer. The frequency of complete or near complete resolution of opthalmoplegia was reported to be 76.5% and the resolution in optic neuropathy was reported to be 75% in the literature [16]. Many pathogenic mechanisms are proposed as the cause of total ophthalmoplegia in zoster infection. These include direct viral cytopathic effect and a reactive immunologic response to the virus [17, 18]. Naumann et al. reported chronic inflammatory cell infiltration in the long posterior ciliary vessels and nerves of 21 enucleated eyes which were affected by HZO [18]. These findings can be suggested by microinfarction of the cranial nerves which was reported previously by Garg et al. [19]. On the other hand, orbital soft tissue edema may affect the third, fourth, and sixth cranial nerves by direct compression [8]. Direct spread of the HZV virus from the fifth cranial nerve to the third, fourth, and sixth in the region of the orbital apex may be one of the other possible mechanisms of total ophthalmoplegia [20]. In our case, we hypnotize that both the compressive effect of the orbital soft tissue edema and the direct spread of the virus caused the total ophthalmoplegia. Also the history of disorientation and syncope in our patient may be due to microinfarction of the brain.

 Orbital apex syndrome is a very rare and severe complication of HZO. Although the effect of systemic steroids and antiviral therapy on HZO-associated ophthalmoplegia has not been studied with a randomised controlled clinical trial, the combination treatment of intravenous acyclovir and steroids usually carries good prognosis especially with normal immunity.
